# The role of incentive mechanisms in promoting forest restoration

**DOI:** 10.1098/rstb.2021.0088

**Published:** 2023-01-02

**Authors:** Anazelia M. Tedesco, Pedro H. S. Brancalion, Michelle L. Hak Hepburn, Khalil Walji, Kerrie A. Wilson, Hugh P. Possingham, Angela J. Dean, Nick Nugent, Katerina Elias-Trostmann, Katharina-Victoria Perez-Hammerle, Jonathan R. Rhodes

**Affiliations:** ^1^ School of Earth and Environmental Sciences, The University of Queensland, Brisbane, QLD 4072, Australia; ^2^ Centre for Biodiversity and Conservation Science, The University of Queensland, Brisbane, QLD 4072, Australia; ^3^ School of Biological Sciences, The University of Queensland, Brisbane, QLD 4072, Australia; ^4^ Centre for the Environment, Queensland University of Technology, Brisbane, QLD 4000, Australia; ^5^ Departamento de Ciências Florestais, Escola Superior de Agricultura Luiz de Queiroz, Universidade de São Paulo, Piracicaba 13418-900, Brazil; ^6^ Department of Anthropology, The University of British Columbia, Vancouver, BC Canada, V6T 1Z4; ^7^ Forestry Division, Food and Agriculture Organization of the United Nations (FAO), Viale delle Terme di Caracalla, Rome 00153, Italy; ^8^ World Agroforestry Centre (ICRAF), United Nations Avenue, Nairobi, 00100, Kenya; ^9^ School of Agriculture and Food Sciences, The University of Queensland, Gatton, QLD 4343, Australia; ^10^ Yale School of the Environment, Yale University, 195 Prospect Street, New Haven, CT 06511, USA; ^11^ BNP Paribas, Katerina Elias-Trostmann, Sustainability and ESG, BNP Paribas, Avenida Presidente Juscelino Kubitschek, 1909, Sao Paulo 04543-907, Brazil

**Keywords:** ecosystem restoration, forest landscape restoration, forest conservation, nature-based solutions, natural regeneration, social-ecological systems

## Abstract

Forest restoration has been proposed as a scalable nature-based solution to achieve global environmental and socio-economic outcomes and is central to many policy initiatives, such as the Bonn Challenge. Restored forests contain appreciable biodiversity, improve habitat connectivity and sequester carbon. Incentive mechanisms (e.g. payments for ecosystem services and allocation of management rights) have been a focus of forest restoration efforts for decades. Yet, there is still little understanding of their role in promoting restoration success. We conducted a systematic literature review to investigate how incentive mechanisms are used to promote forest restoration, outcomes, and the biophysical and socio-economic factors that influence implementation and program success. We found that socio-economic factors, such as governance, monitoring systems and the experience and beliefs of participants, dominate whether or not an incentive mechanism is successful. We found that approximately half of the studies report both positive ecological and socio-economic outcomes. However, reported adverse outcomes were more commonly socio-economic than ecological. Our results reveal that achieving forest restoration at a sufficient scale to meet international commitments will require stronger assessment and management of socio-economic factors that enable or constrain the success of incentive mechanisms.

This article is part of the theme issue ‘Understanding forest landscape restoration: reinforcing scientific foundations for the UN Decade on Ecosystem Restoration’.

## 1. Introduction

Forest restoration is a promising nature-based solution for addressing the critical challenges of the Anthropocene [1]. To help move restoration efforts to scale and accelerate political action against ecosystem degradation, the United Nations declared 2021 to 2030 the ‘Decade on Ecosystem Restoration’ [2]. This declaration builds upon years of progress in stimulating forest restoration through a diversity of incentive mechanisms targeting landholders and land users. These mechanisms include financial incentives, such as payment for ecosystem services (PES) or non-financial forms of institutional support, such as technical assistance, and are defined as ‘instruments used by the public and private sectors to encourage farmers to protect or enhance ecosystem services beneficial to them and others' [3]. These mechanisms have promoted sustainable practices in natural resource management and conservation in private land [4,5]; yet knowledge about their role in achieving successful restoration remains limited. Restoration interventions include a broad range of strategies such as natural forest regrowth (passive restoration), agroforestry and mixed plantations of native species (active restoration), each with specific enabling or constraining conditions that influence specific restoration outcomes. These interventions have varying scales and timelines, ranging from short-term interventions on small parcels of land to large-scale, holistic approaches of forest landscape restoration (FLR). A better understanding of the enablers and constraints of restoration incentives is critical for scaling up efforts to meet global and regional restoration commitments.

To drive restoration at scale, a plethora of multilateral environment agreements have spawned over the past decade. These started with the Aichi target 15 of the Convention on Biological Diversity (CBD), setting, in 2010, a goal to restore 15% of degraded ecosystems [6], followed by the launch of the Bonn Challenge in 2011. Aiming to restore 350 million hectares of deforested and degraded landscapes by 2030 [7], this challenge inspired regional efforts such as the African Forest Landscape Restoration Initiative (AFR100) and the 20 × 20 Initiative in Latin America [8]. Additionally, the Sustainable Development Goals included reversing land degradation through restoration as a fundamental activity (United Nations 2015 [9]). In 2015, these and other commitments culminated with the Paris Climate Change Agreement [10], further recognizing restoration as an important nature-based solution to the climate crisis. As of 2020, 130 out of 168 Nationally Determined Contributions (77%) have quantitative and/or qualitative FLR-aligned targets [11].

Despite the globally recognized benefits of achieving restoration at scale, doing so cost-effectively remains a challenge [12]. Governments, the private sector and not-for-profit organizations face further barriers, which include financial constraints, limited understanding of the social motivations for restoration and unsuitable governance structures [13–15]. Under these circumstances, the slow progress reported by countries towards meeting ambitious restoration targets raises serious questions about the feasibility of achieving global commitments [16,17]. Even where initial constraints are overcome, ensuring the long-term success of restoration remains challenging [18]. For example, although recent studies in the Brazilian Atlantic Forest demonstrate that it is possible to achieve conservation targets by combining forest restoration and compensation [19], a forest cover change analysis in the same region found that 27% of regenerating forests were recut between 1990 and 2017, particularly in landscapes with high opportunity costs for shifting from agriculture to restoration (e.g. flat fertile terrain preferred for agriculture) [20]. This highlights the critical role that socio-economic factors play in forest restoration success and the influence of incentive mechanisms on this success [21–23].

Ideally, incentive mechanisms should compensate landholders and land users for the foregone opportunities associated with active restoration and natural regrowth in agricultural landscapes. Incentives, such as PES and technical assistance, are also widely used to promote the adoption of sustainable agricultural practices [3] and can be classified as direct or indirect incentives. Direct incentives are designed to have a direct impact on resource users (e.g. PES), whereas indirect incentives have an indirect effect by setting or changing the overall conditions in which land is managed (e.g. technical support or management rights) [24]. If used to combine restoration and income generation (e.g. through timber production and the adoption of agroforestry systems), incentive mechanisms can contribute to both ecological outcomes and socio-economic change, playing an important role for local livelihoods [25]. Due to the prospect of socio-economic benefits, these strategies seek to motivate engagement of landholders in restoration [26], compared to approaches that solely stimulate restoration through legal or governance mechanisms. Yet, enhancing motivation is not sufficient to guarantee engagement and success; the social–ecological context of these incentive programs is likely to be an important factor that facilitates the capacity and opportunity to engage in restoration, and supports positive ecological and socio-economic outcomes [27,28].

Here, we conduct a systematic literature review to address three main research gaps: (i) What types of incentive mechanisms are used for forest restoration and how are these financed? (ii) What biophysical and socio-economic factors affect the success of incentive mechanisms to promote forest restoration? (iii) What have been the ecological and social outcomes of incentive mechanisms for promoting forest restoration? On the basis of our findings, we discuss the challenges and potential of using incentive mechanisms as pathways to promote large-scale, long-term restoration, while maximizing ecological and social benefits.

## 2. Methods

### (a) Literature search

We conducted a literature review of peer-reviewed articles published between January 2010 and October 2020. This time frame was selected to understand the current trends in incentive mechanisms for forest restoration, as preliminary literature searches showed that few papers on incentives for restoration were published before 2010, compared to more recent years, with a strong focus on the Reducing Emissions from Deforestation and Forest Degradation (REDD+) incentives. We also identified that few studies have examined the role of disincentive mechanisms, such as penalties, fines and quotas. For this reason, we decided to focus on incentives for this literature review. The literature search was conducted via: (i) database search using key terms; (ii) screening of studies resulting from the initial search; and (iii) selection of papers for final analysis based on eligibility criteria. Key words searched include incentive mechanism terms (first filter) and forest restoration and regrowth terms (second filter), such as ‘payment* for ecosystem services’ and ‘ecological restoration’, respectively. Electronic supplementary material, figure S1 details the selection of studies for the analysis in a PRISMA diagram. A detailed description of the literature search, search terms and paper selection is also provided in the electronic supplementary material (electronic supplementary material, methods). Searches returned 1421 papers that were imported to the software Covidence [29]. After the exclusion of 312 duplicates, four of the authors (A.M.T., M.L.H.H., K.W. and N.N.; henceforth ‘coders’) screened the 1109 remaining studies based on title and abstract. Each study abstract was screened by two authors independently to select those that (i) consisted of an assessment or application of an incentive mechanism; (ii) indicated that the intervention aimed to promote forest restoration; and (iii) included information about on-ground interventions (i.e. modelling of hypothetical incentives were not considered). The interventions considered encompass a broad range of approaches defined as forest landscape restoration (FLR) strategies [30], as follows: (i) active forest restoration (native tree planting or direct sowing); (ii) afforestation (establishment of tree cover on lands that, historically, have not contained forests [31]; (iii) sustainable practices (agroforest plantation or silvopastoral system adoption); (iv) natural forest regrowth (the regrowth of native forests through natural regeneration); and (v) sustainable forest management or forest conservation (only considered here when incentivized in combination with one of the previous interventions aimed at promoting forest restoration). Finally, when Covidence indicated discrepancies between coders, the study in question was further reviewed until authors reached consensus on the papers that met the eligibility criteria. In a second round of screening, the 217 papers that fulfilled the criteria were assessed in full to confirm eligibility, reducing the final number of articles included in the analysis to 73.

### (b) Data analysis

To answer the first research question, through full-text reading of each paper and using 15 structured questions (electronic supplementary material, table S1), we gathered descriptive data on three main aspects of the evaluated incentive mechanism: (i) general characteristics: country, time frame of intervention at the time of evaluation, spatial scale of implementation, land-tenure regimes in the area of implementation and primary land use; (ii) governance characteristics: type of proponent organizations, type of incentive, type of financing mechanism, source of funding, type of governance and targeted recipients; and (iii) the restoration approaches incentivized (e.g. active forest restoration and agroforest plantation).

To answer the second and third research questions, we conducted a thematic analysis using NVivo software [32]. We used a combined deductive/inductive approach [33] to code information related to outcomes of the incentive being applied, as well as to factors affecting implementation success of the initiatives. Both outcomes and factors affecting implementation success were considered as they were defined in the studies, which varied across diverse timescales, depending on the interest of the study or on the different objectives of the incentives implemented (e.g. poverty alleviation or carbon sequestration). Methods used by the studies also varied between qualitative, quantitative or mixed methods approaches. To conduct the thematic analysis, we inductively coded outcomes and factors affecting implementation success within predefined categories. These children codes emerged from the results presented in each study. We then revised and grouped the children codes into themes for interpretation purposes, maintaining the original predefined categories. For factors affecting implementation success—defined here as the attainment of the incentive's objectives (e.g. forest cover increased or biodiversity enhanced), as described in the reviewed papers—the main predefined categories were ‘Enablers’ and ‘Constraints’. Within these, we classified factors as ‘Biophysical’, ‘Social’ (governance structures, cultural norms and individual characteristics) or ‘Economic’ (financial conditions). Similarly, we coded outcomes of incentive mechanisms under ‘Beneficial outcomes’ (positive impacts reported as results of an incentive mechanism), ‘Unmet objectives’ (unattained desired outcomes of an incentive mechanism) and ‘Perverse outcomes’ (unintended negative impacts reported as results of an incentive mechanism). Within those categories, outcomes were organized into ‘Ecological’ and ‘Socio-economic’. Each outcome was determined based on the original study included in the review. ‘Ecological outcomes’ reflected biophysical changes resulting from the incentive mechanism implementation (e.g. forest cover increase and water quality improvement), whereas ‘Socio-economic outcomes’ reflected social and economic changes resulting from the incentives (e.g. livelihood improvement and income generation). For example, perverse ecological outcomes were classified into ‘Reduced ecosystem services or natural capital’ (e.g. reduction of species diversity and decrease in forest cover) and ‘Spillover and non-permanence’ (e.g. reconversion to farming), whereas perverse socio-economic outcomes, for instance, were coded into ‘Governance’ (e.g. the reduction of engagement), ‘Inequity’ and ‘Livelihood assets reduced’ categories. The complete set of codes is available in the electronic supplementary material.

## 3. Results

### (a) Incentive mechanisms for forest restoration

**Figure 1.**
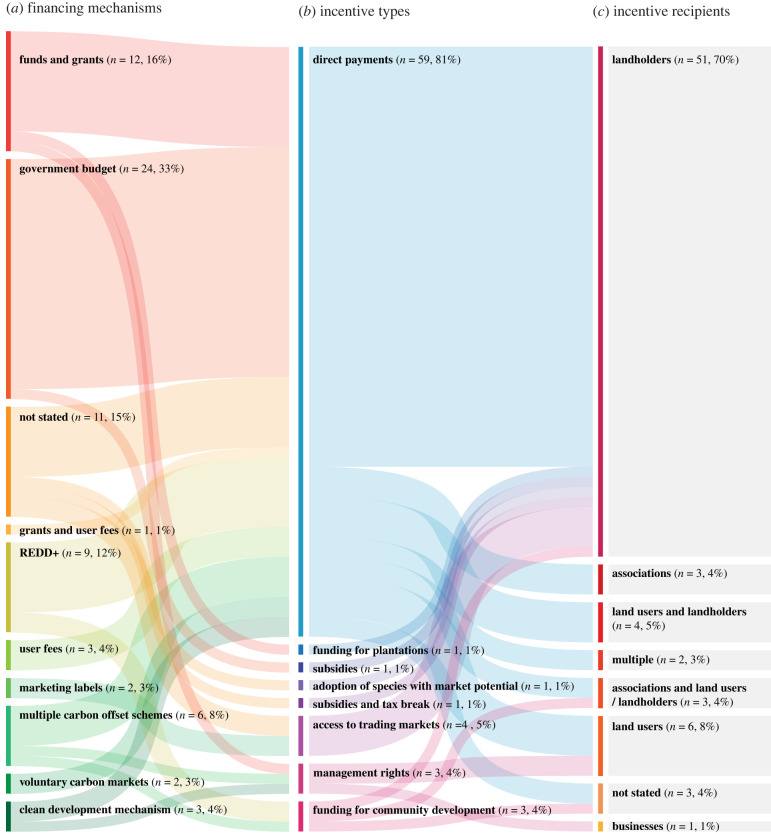
Links between (*a*) financing mechanisms, (*b*) incentive types and (*c*) incentive recipients. This shows the proportion of studies mentioning each source of funding or financing mechanisms (left side) supporting the primary incentive assessed in the studies (centre); and the proportion of each incentive mechanism targeting each type of recipient (right side). Links are colour-coded by source and the figure is read left to right. The financing mechanism class labelled ‘multiple carbon offset schemes’ combines REDD+, clean development mechanism and voluntary carbon markets. Institutional or in-kind support and tax breaks were offered only in combination with incentives and were not included in this figure. (Online version in colour.)

**Figure 2.**
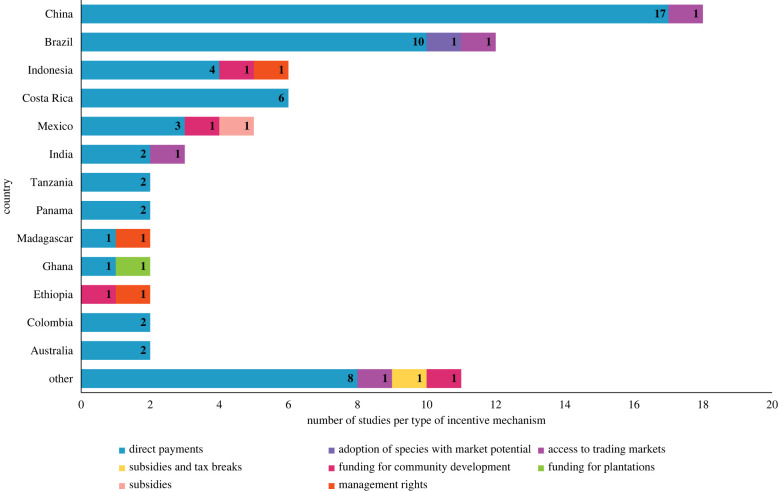
Incentive mechanisms were trialled in each country covered by the reviewed papers. Numbers indicate the frequency of each type of incentive among assessed studies. (Online version in colour.)

In total, 73 papers met the eligibility criteria (electronic supplementary material, table S2), with 25 (34%) of these published since 2019 (electronic supplementary material, figure S2*c*). This increasing publication rate reflects the growing interest in incentive mechanisms and forest restoration. The publications covered 10 different types of incentives, offered alone or in combination (figure 1; electronic supplementary material, figure S2*a*), across 24 countries (figure 2). Electronic supplementary material, table S2 details the countries covered by each paper, as well as the focus of each study (i.e. factors affecting implementation or outcomes of incentives offered). Apart from four studies focused on incentives applied in Australia, Ireland and the United States, all papers covered incentives offered in countries in the Global South. Among those, China and Brazil were the most referenced regions, with 18 and 12 papers related to incentives in these countries, respectively. There was a clear predominance of studies focusing on direct payments as an incentive for forest restoration (59 studies, 81%), with other incentive types covered by five (7%) or fewer studies each. Tax breaks and institutional or in-kind support were only offered in combination with other mechanisms. Active forest restoration (i.e. based on native tree planting or direct sowing) was the most common implementation approach (47 studies, 64%; electronic supplementary material, figures S2*e* and S3), followed by forest conservation or sustainable forest management (always combined with a restoration approach, in projects incentivising multiple strategies; 32 studies, 44%) and adoption of sustainable land use practices (27 studies, 37%). Natural forest regrowth and afforestation (establishment of tree cover on lands that historically have not contained forests; [31]) were the least incentivized restoration approaches (20 studies, 27%; and 18, 25% respectively). Most of the studies (57%) evaluated initiatives that had been in operation for more than 6 years by the time of the assessment (electronic supplementary material, figure S1*b*) and were implemented at a spatial scale of 1000 km^2^ or greater (58%; electronic supplementary material, figure S1*d*).

We found that the types of incentive mechanisms implemented varied according to some governance characteristics at the plot and landscape scale, such as the target area's primary land use and the incentive's institutional governance type (electronic supplementary material, figure S4). At the plot scale, more than 65% of the direct payments incentivized restoration where agricultural production was the primary land use (electronic supplementary material, figure S4*a*). By contrast, for other types of incentive mechanism, only 29% of the studies involved agricultural production as the primary use. At the landscape scale, overall, public-led governance was the most common mechanism among the reviewed studies (65%). The public sector was the proponent (the organization responsible for the implementation) for 64% of initiatives offering direct payments (electronic supplementary material, figure S4*c*). Similarly, there was a dominance of public-led initiatives among the other incentive types (71%). For non-public-led initiatives, such as third-sector (e.g. charities, philanthropic organizations) and private-led initiatives, the incentives were almost exclusively direct payments, except for one third-sector initiative using access to trading markets as an incentive for forest restoration. On the other hand, land-tenure arrangements did not influence the type of incentive mechanisms applied. Private land-tenure regimes were the most frequent among the studies, for both direct payments (38% of studies) and all other incentives considered together (36%), while other tenure arrangements were broadly distributed among all incentive types (electronic supplementary material, figure S4*b*).

A variety of financing mechanisms operationalized incentives for forest restoration (figure 1). Almost one-third of the papers (33%) cited government budgets as the main source of funding. Carbon offset schemes (e.g. Clean Development Mechanism, REDD+, and voluntary carbon markets, alone or in combination) accounted for 26% of the financing of forest restoration incentives. Other financial sources included funds and grants (16%), fees applied to ecosystem services users (4%; e.g. water user fees imposed to companies) and marketing labels (3%). While many types of beneficiaries were targeted by the incentives, we found that most recipients were individual or communal landholders (70%), receiving the largest share of direct payments. Only 17% of incentive recipients fell under categories that included land users.

### (b) Factors affecting implementation success

**Figure 3.**
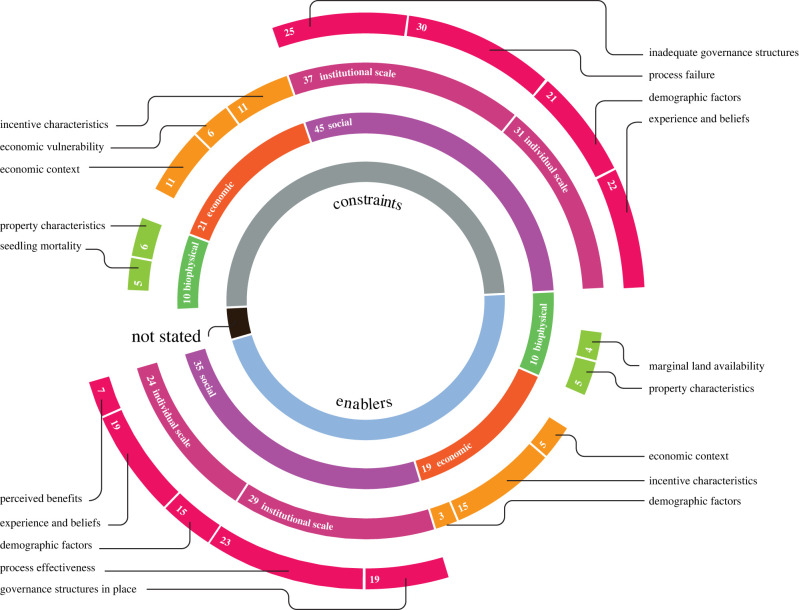
Factors affecting implementation success of incentive mechanisms for forest restoration. Numbers indicate the frequency of each factor among assessed studies. (Online version in colour.)

Our review revealed that social factors—including incentive governance and target beneficiary characteristics—were the most commonly reported influences on program success. A broad range of socio-economic factors were associated with the success or failure of incentive mechanisms for forest restoration (figure 3, table 1). The majority of the studies (61%) indicated social factors as constraints for implementing incentives. Economic factors (28% of studies) and biophysical aspects (13%) were less frequently mentioned as constraints. When assessing factors leading to the success of initiatives, the studies also identified social factors (48% of papers) more often than economic (26%) and biophysical aspects (13%).

**Table 1.** Themes and examples (non-exhaustive list) of categories mentioned in the studies as factors affecting implementation and long-term success of incentive mechanisms for forest restoration. The complete set of codes is available in the electronic supplementary material.

constraints		
*social*		
institutional scale	inadequate governance	legal uncertainty
		lack of financial support
		lack of capacity-building and extension services
	process failure	bureaucratic participation process
		late payments
		short duration of incentives
individual scale	experience and beliefs	lack of interest
		social participation
		low labour availability
	demographic factors	gender
		level of education
*economic*		
economic context	change in carbon prices	
	competing economic activities	
incentive characteristics	evidence-based payments (after restoration)	
	small payments	
*biophysical*		
property characteristics	challenging topography	
	remoteness	
	small size of area available	
	susceptibility to climate hazards	
project execution	high-seedling mortality rate	
enablers		
*social*		
institutional scale	governance structures in place	adequate legal framework
		political will
		partnership among stakeholders
	process effectiveness	non-bureaucratic agreements
		effective monitoring
		conflict management
individual scale	experience and beliefs	existing social networks
		legislation compliance
		existing diversified livelihoods
	perceived benefits	access to training
		previous positive experiences
		perceived ancillary benefits
	demographic factors	place of residency and land use
		level of education
*economic*		
economic context	profitability of alternative land use offered	
	adequate access to markets	
incentive characteristics	offer of alternative livelihoods	
	sufficient financial incentive	
participant's characteristics	access to capital	
	diversified income source	
	economic vulnerability	
*biophysical*		
property characteristics	marginal land availability	
	low altitudes	

Constraints at the institutional scale included communication issues (11 studies, 15%), lack of beneficiary engagement (7, 9%), legal uncertainty (7, 9%), weak participatory component in the decision-making (9, 12%) and lack of transparency (5, 7%). Late payments and short duration of incentives were also mentioned in some cases (3 studies, 4%). At the individual scale, constraints consisted of, among other aspects, lack of access to land or to markets (18 studies, 25%), socio-cultural values or norms related to the traditional use of land (9, 12%) and scepticism or lack of trust in institutions involved in the project governance (6, 8%). The most frequent constraints related to the economic context and incentive characteristics were competing economic activities (5 studies, 7%) and small payments offered (6, 8%), respectively. Biophysical constraints were less frequently identified and included property characteristics such as small area size available and susceptibility to climate hazards (2 studies, 3%, each).

We found that enablers at the institutional scale included partnerships among stakeholders (12 studies, 16%), appropriate legal frameworks (8, 10%) and non-bureaucratic agreements (6, 8%). Enabling factors at the individual scale included motivational aspects such as the perceived benefit of planting trees (3 studies, 4%), social norms and values influencing decisions (5, 7%) and access to information and level of education (7, 9%). Commonly, the same factors were mentioned as enablers and constraints in different studies, depending on the implementation context (e.g. adequate legal frameworks versus legal uncertainty, effective communication versus inadequate communication, sufficient payments versus small payments).

### (c) Outcomes of incentive mechanisms for forest restoration

**Figure 4.**
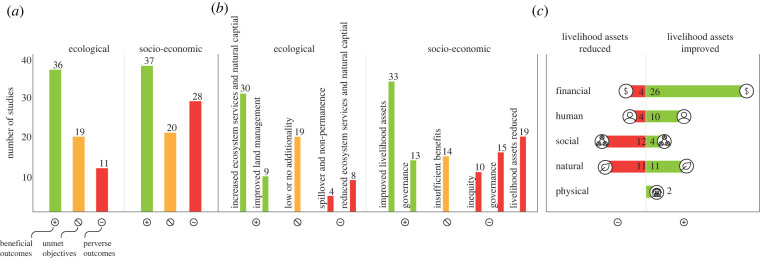
Outcomes of incentive mechanisms for forest restoration. Numbers indicate the frequency of each factor among assessed studies: (*a*) overall number of studies that reported beneficial outcomes, unmet objectives, and perverse ecological and socio-economic outcomes; (*b*) number of studies for each category of beneficial outcomes, unmet objectives, and perverse ecological and socio-economic outcomes; (*c*) number of studies reporting impacts in each kind of livelihood asset. In (*b*) columns sum to more than the total number shown in (*a*), and in (*c*) numbers sum to more than the total number of improved and reduced livelihood assets in (*b*) since more than one type of outcome can be reported by the same study. (Online version in colour.)

Out of the three analysed outcomes resulting from incentive mechanisms (beneficial, perverse and unmet outcomes), beneficial outcomes dominated reporting for both ecological (reported in 49% of studies) and socio-economic aspects (50%) (figure 4*a*). A similar proportion of unmet objectives were identified for ecological (in 26% of studies) and socio-economic aspects (27%). Finally, perverse outcomes were more commonly reported for socio-economic aspects (38% of studies) than ecological (15%). These ecological and socio-economic outcomes were summarized within five and six main classes, respectively (figure 4*b*).

The most mentioned beneficial ecological outcomes were increased ecosystem services and natural capital, cited in 41% of studies. Within those, increased forest cover (29%), carbon sequestration (7%) and improved water quality and availability (7%) are among the most frequent outcomes reported. Studies reporting low or no additionality of incentives (i.e. no differential benefit) to ecological aspects (26%) include low additionality to the provision of ecosystem services or natural capital (11%), and lack of evidence of effectiveness of restoration incentives to ecological impacts (11%). Perverse ecological outcomes were linked to spillover (negative effects outside of the area of intervention; 5%) and reduced ecosystem services (11%).

**Figure 5.**
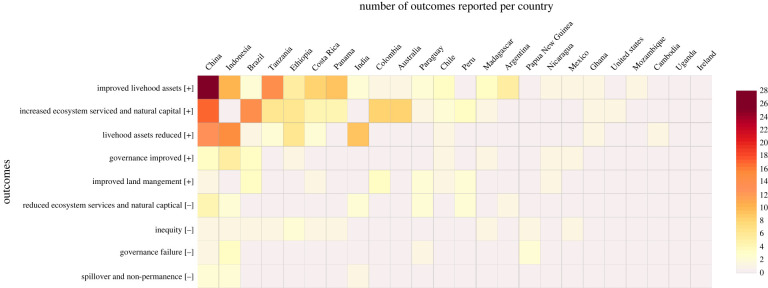
Number of positive [+] and negative [−] outcomes reported across countries in the studies assessed. (Online version in colour.)

Among the socio-economic factors, governance beneficial outcomes were reported in 18% of the studies, with increased stakeholder engagement (8%) being the most mentioned outcome. Insufficient benefits for income generation (12% of studies) and unfulfilled participants’ expectations (15%) were the primary unmet socio-economic objectives. Perverse governance outcomes and inequity outcomes included reduction of engagement (one study) and unequal distribution of benefits (8 studies, 11%), respectively. The beneficial and perverse outcomes of incentives for livelihoods assets (cited in 45% and 26% of the studies, respectively) are detailed in figure 4*c*. Figure 5 details the distribution of positive and negative outcomes across the countries covered by the studies assessed.

## 4. Discussion

Understanding how incentive mechanisms are used for promoting forest restoration and what constrains and enables their success is critical both for upscaling restoration to meet international commitments and achieving multiple targeted benefits. We found that direct payments (e.g. PES), financed mainly through government budgets, carbon offset mechanisms and funds and grants, were the most commonly described incentive mechanisms for forest restoration. In addition, we found that constraints for implementation and long-term success of incentives for restoration are predominantly socio-economic and governance-related, rather than ecological or biophysical. Although ecological factors have been mostly used to assess restoration success [34,35], we found them to be the least frequently mentioned constraints or enablers for forest restoration incentives. This is consistent with findings for incentives for biodiversity conservation and natural resource management practices [36–38]. Our review indicates that the major challenges to improve incentive mechanisms that promote long-term forest restoration, are (i) adequately accounting for the integration of ecological and socio-economic aspects, (ii) using incentives to create a restoration value chain that does not rely on financial incentives in the long term and (iii) considering the variety of incentive mechanisms that can be used in different contexts to achieve the greatest long-term social and ecological benefits.

### (a) Integrating ecological and socio-economic research and practice

We found that ecological aspects were rarely mentioned as constraints or enablers for the implementation of restoration incentives. This is surprising, since ecological constraints of restoration are extensively documented in the literature [39]. They often reflect the degradation of project sites and can range from unsuitable substrates to low propagule availability, including factors such as invasive species and poor survival of seedlings due to drought and herbivory [39,40]. These biophysical aspects have been addressed in the planning, implementation and monitoring of restoration for several decades, and could reflect the maturity of restoration projects. Further, the academic literature on ecosystem restoration, largely dominated by ecological studies, poorly covers the socio-economic aspects of restoration [35,41], contrasting with the findings of our review. Thus, there is a mismatch between restoration research and incentives research. This is a critical barrier for scaling up restoration to achieve global commitments and ensuring that incentives mechanisms can deliver on outcomes.

While most of the studies included in our review evaluated incentive outcomes, only a few used control groups as part of the research design [42–44]. This reveals a need for better monitoring and evaluation of incentive mechanisms and applying standardized approaches over adequate time frames. Stronger quantitative evidence documenting long-term ecological and socio-economic outcomes and ingredients for success [45,46] could assist organizations in selecting the most appropriate incentive types to achieve the greatest long-term social and ecological benefits per dollar invested, similarly to investment decisions regarding different restoration approaches (e.g. active versus passive restoration; [47,48]). Ideally, this knowledge would be integrated into an adaptive management framework to facilitate learning from failures and successes over time.

### (b) Integrating social aspects into restoration planning—implications for restoration incentives

While the theoretical frameworks for integrating social and ecological systems have been extensively discussed [49–51], translating this integration to practice remains a challenge. A growing body of research has recognized the importance of considering socio-economic aspects in restoration and conservation planning, both as objectives and constraints [52–56]. Nevertheless, many restoration prioritization analyses are still largely based on biophysical factors, with socio-economic aspects—which are less mappable—often restricted to only considering opportunity costs [57,58]. Our review highlights how opportunity costs, alone, provide insufficient explanation for uptake or success of incentive mechanisms. Identifying areas with a high chance of long-lasting restoration success may require navigating through social factors, such as power relationships, land-tenure and stakeholder engagement [59,60]. For example, many of the social factors contributing to failure, such as lack of beneficiaries’ engagement and weak participatory components [61], may be linked to conflicting goals between stakeholders [62]. While local actors—often the main targets of incentive mechanisms—are usually interested in farm-scale outcomes (e.g. improved water quality and availability), organizations that lead or finance the implementation of these instruments are often interested in regional- and global-scale outcomes, such as landscape connectivity and climate change mitigation [63]. As such, applying incentives in restoration priority areas selected based on ecological factors will require ‘social ground truthing’ to explore and better understand local social dynamics, restoration supply chains, leadership and political will [64]. Similarly, organizations financing or leading the implementation of incentive mechanisms for restoration may want to balance their efforts by prioritizing actions not only across space, but also over time. Thus, they could take advantage of existing governance structures to begin restoration implementation while supporting the development of adequate governance and local institutional capacities in other priority areas where these do not already exist.

### (c) Financing incentive mechanisms

Similarly to ecological aspects, the lack of funding is widely recognized as a crucial constraint for restoration [65] but was cited as a constraint for only 5% of the studies. It is possible that this aspect may have been overlooked in the studies included in our review, as we included only on-the-ground studies on initiatives already implemented or under implementation; potential restoration projects that were not implemented due to insufficient funds were likely not to be reported in the literature and represent a bias in our study. Moreover, the scientific literature on incentives still does not reflect current trends in restoration financing. Funding mechanisms supporting the incentives captured in our review (e.g. government budget, carbon offset mechanisms) have been traditionally used in restoration funding. Globally, however, an increasing number of innovative initiatives aim to unlock public and private financial resources for restoration [66]. These innovative initiatives include new finance and partnership platforms that offer access to pipelines of investment opportunities. With the promise of linking socio-economic development and conservation, such strategies strongly envision social impact with a financial return. Yet, the incentive mechanisms supported by these initiatives will need to consider underlying socio-economic factors such as those assessed in our review to ultimately attain their multiple objectives.

### (d) Using incentives to create restoration value chains

It is crucial to use incentive mechanisms to develop a restoration value chain that translates into long-term financial and livelihood gains, increasing the value of standing forests within agricultural landscapes. As found in our review, this is important because agriculture is often the primary land use in areas targeted by restoration incentives. Many restoration programs offer short-term payment contracts for promoting long-term land use changes. This creates a critical temporal gap between restoration incentives and outcomes, which may undermine landholders’ and users’ confidence in restoration's financial viability [67]. Socio-economic constraints related to income could be overcome by using incentives in a locally relevant manner (e.g. by offering alternative livelihoods or promoting sustainable land uses already in place), yet the long-term sustainability of such incentive approaches remains a critical issue to consider. Our review demonstrated that inadequate payments, or short duration of financial incentives, contribute to implementation failure, and may eventually lead to reconversion of restored areas to agro-pastoral land uses [68]. However, if the incentive targets an approach that proves to increase farmer livelihoods (e.g. silvopastoral systems adoption), it is possible that even short-term direct payments promoting tree cover in agricultural landscapes can lead to persisting benefits [69]. Another promising restoration strategy is intercropping exotic and native timber species to create financially viable restorative systems designed to offset implementation costs. In these systems, the exploitation of fast-growing exotic trees in short rotation offers long-term, continued returns from native timber harvesting while still providing favourable conditions for natural regeneration [70]. Incentive mechanisms able to promote these strategies and function as an ignition point to restoration value chains could be discontinued once these structures are in place, without risking reconversion of restored areas to alternative land uses. This would promote effective use of limited available funding in face of the massive global restoration targets.

### (e) Adapting incentives to local contexts

No incentive type is universally suitable for all socio-economic contexts; as such, mechanisms to promote restoration should be selected based on local needs and context. Our review revealed the use of diverse incentives, likely to attract different target groups. These included, for example, direct payments (both for ecosystem services and adoption of sustainable practices), funding for community development and allocation of management rights [71–73]. Consistent with research about the uptake of incentive mechanisms [74], there was a clear contextual influence on the type of incentive applied. For example, the high percentage of direct payments occurring in areas where agricultural production is the primary use is likely linked to the fact that these areas are mostly privately owned. In this context, direct payments are probably seen as the most advantageous incentive by landholders, as they often offer prompt compensation for the income lost from reconversion of agro-pastoral lands to forests. Alternatively, incentives can promote access to markets or adoption of species with market potential. Overcoming land opportunity costs may be a prerequisite for successful restoration in contexts where other aspects, such as legislation obliging restoration or certification demands [19], are not in place.

Conversely, mechanisms that incentivize restoration by offering land use management rights or alternative livelihoods could appeal more to land users or landholders with informal land-tenure. Despite a dominance of direct payments in our review, it remains unclear to what extent the papers selected simply reflect prevailing research and funding interests or an accurate proportion of types of incentive mechanisms implemented. Additionally, there may be a bias against non-economic incentives, such as institutional support and the offer of extension services. Frequently, these activities are assumed to be ubiquitous and could be underrepresented in the literature on incentives. Nonetheless, the heterogeneity of incentive mechanisms reinforces the need to engage with stakeholders early in the design and implementation process, to ensure that incentive schemes align well with their needs, overcome constraints and safeguard the critical enabling conditions for successful restoration.

## 5. Conclusion

Although incentive mechanisms, especially when implemented by governments, are essentially seen as policy levers with a strong focus on finance and payment, the original aim of these instruments is to create system-wide change [75,76]. When applied to stimulate restoration, these mechanisms are usually part of a complex web of mechanisms and policies that contribute to the longevity of restored ecosystems [18]. Therefore, their implementation should consider the complexity of the coupled socio-ecological system in which restoration occurs. In our review, we found that socio-economic factors, such as governance, monitoring systems and the experience and beliefs of participants, dominate whether or not an incentive mechanism is successful. Moreover, we found that while approximately half of the studies report both positive ecological and socio-economic outcomes from incentives for forest restoration, adverse outcomes were more commonly socio-economic than ecological. Our results reveal that achieving forest restoration at a sufficient scale to meet international commitments will require stronger assessment and management of socio-economic factors that enable or constrain the success of incentive mechanisms. Fundamentally, restoration incentive mechanisms should be seen as a means to attain the multiple objectives of restoration [77–79] and even initiatives primarily aimed at achieving ecological outcomes should integrate social and economic aspects in their design and implementation [50,80,81]. A broader multi-disciplinary approach is crucial to overcome social and economic constraints, to avoid perverse socio-economic outcomes and to maximize long-term benefits for people and nature.

## Data Availability

Data are available from UQ eSpace repository: https://doi.org/10.48610/c1e51da [82]. The data are provided in the electronic supplementary material [83].
